# Effects of non-invasive brain stimulation combined with cognitive training on cognitive functions in older people with mild cognitive impairment: a systematic review with meta-analysis

**DOI:** 10.3389/fmed.2025.1659208

**Published:** 2025-10-09

**Authors:** Edgar Vásquez-Carrasco, Pía Jamett-Oliva, Amanda Quijada, Jordan Hernandez-Martinez, Braulio Henrique Magnani Branco, Eduardo Carmine-Peña, Paulina Sepúlveda, Cristian Sandoval, Pablo Valdés-Badilla

**Affiliations:** ^1^School of Occupational Therapy, Faculty of Psychology, Universidad de Talca, Talca, Chile; ^2^Centro de Investigación en Ciencias Cognitivas, Faculty of Psychology, Universidad de Talca, Talca, Chile; ^3^VITALIS Longevity Center, Universidad de Talca, Talca, Chile; ^4^Department of Physical Activity Sciences, Universidad de Los Lagos, Osorno, Chile; ^5^Graduate Program in Health Promotion, Cesumar University (UniCesumar), Maringá, Brazil; ^6^Carrera de Medicina, Facultad de Medicina, Universidad de La Frontera, Temuco, Chile; ^7^Departamento de Ciencias Preclínicas, Facultad de Medicina, Universidad de La Frontera, Temuco, Chile; ^8^Escuela de Tecnología Médica, Facultad de Salud, Universidad Santo Tomás, Osorno, Chile; ^9^Departamento de Medicina Interna, Facultad de Medicina, Universidad de La Frontera, Temuco, Chile; ^10^Department of Physical Activity Sciences, Faculty of Education Sciences, Universidad Católica del Maule, Talca, Chile; ^11^Sports Coach Career, Faculty of Life Sciences, Universidad Viña del Mar, Viña del Mar, Chile

**Keywords:** aged, cognition, older people, rehabilitation, technology

## Abstract

**Background:**

Non-invasive brain stimulation (NIBS) has emerged as a potential adjunct to cognitive training for enhancing cognitive performance in older peoples with mild cognitive impairment (MCI). This systematic review and meta-analysis aimed to evaluate the efficacy of combined NIBS and cognitive training on cognitive function in this population.

**Methods:**

A comprehensive literature search was conducted in PubMed, EBSCOhost, CINAHL Complete, Cochrane Library, ProQuest, Scopus, and Web of Science up to May 2025. The review followed PRISMA guidelines, and methodological quality was assessed using the Oxford Centre for Evidence-Based Medicine levels, the Cochrane Risk of Bias 2 tool (RoB 2) tool for risk of bias, and the Grading of Recommendations, Assessment, Development and Evaluation (GRADE) approach for the certainty of evidence. The protocol was registered in PROSPERO (CRD42024563219). Studies were included if they assessed the effects of NIBS in combination with cognitive training on cognitive outcomes in older peoples with MCI.

**Results:**

A total of 1,689 records were screened, and 10 studies met the inclusion criteria. The results indicated a moderate positive effect of the combined intervention on attention and processing speed as measured by the Trail-Making Test Part A (TMT-A; effect size = 0.54). Improvements were also observed in global cognition as assessed by the Montreal Cognitive Assessment (MoCA), though the results were not statistically significant (*p* > 0.05). No significant effects were found for the Trail-Making Test Part B (TMT-B), with effect sizes ranging from 0.05 to 0.52.

**Conclusion:**

The combination of NIBS and cognitive training appears to yield beneficial effects on specific cognitive domains, particularly attention and processing speed, in older people with MCI. These findings support the potential role of NIBS as an adjunctive intervention to cognitive training for enhancing cognitive function in this population. Further high-quality randomized controlled trials are warranted to confirm these effects.

**Systematic review registration:**

https://www.crd.york.ac.uk/PROSPERO/view/CRD42024563219, identifier (CRD42024563219).

## 1 Introduction

The World Health Organization warns that population aging is accelerating worldwide, with the proportion of individuals aged 60 years and older expected to rise from 12% in 2015 to 22% in 2050, and nearly 80% of them living in low- and middle-income countries (1). Aging is closely associated with a higher prevalence of chronic conditions, including cognitive decline, which often precedes dementia (2). Within this spectrum, mild cognitive impairment (MCI) is a clinical condition characterized by measurable deficits in cognitive function that exceed normal age-related changes but do not yet meet the criteria for dementia (3, 4). Recent epidemiological data indicate that MCI affects 15–24% of older adults, with incidence rates increasing to 60 per 1,000 person-years after age 85 (5). Moreover, the global population aged 65 years and older is projected to grow from 771 million in 2022 to 1.6 billion by 2050, underscoring the urgent need for strategies that promote healthy aging and strengthen health systems to meet this demographic challenge (6). A study by Prince et al. (7) indicates that, worldwide, 23% of healthcare expenditure is allocated to the treatment of diseases in people over 60 years of age, and 7% of that expenditure corresponds to neurological and mental disorders, among which MCI is included. Various factors influence aging, such as health, autonomy, cognitive function or capacity, and quality of life (8, 9). Thus, within older people, there is a group that experiences healthy aging and another that is affected by various pathologies (10). Among the conditions associated with aging is MCI, which is defined as a decline in memory or other cognitive functions greater than expected for a person’s age and educational level (11). MCI is characterized by cognitive impairment that does not significantly interfere with basic or instrumental activities of daily living (12).

In recent years, interest in therapeutic interventions for MCI has grown significantly because of the high risk of progression to dementia (13). Individuals with MCI therefore represent an ideal clinical group for testing and developing therapeutic strategies during the early stages of disease progression (14). Ayala San Martín (15) identifies several protective factors that enhance cognitive performance and help delay the onset of dementia. Among the most evidence-based interventions for preventing or slowing the progression of MCI is the combination of physical activity and cognitive stimulation, which promotes health and reduces disease risk, particularly in healthy older peoples (16, 17). Current evidence supports their use as key strategies to improve both cognitive and physical function in this population (18).

Recent interventions have provided substantial evidence for the use of technology-based approaches, such as non-invasive brain stimulation (NIBS), which includes electrical, magnetic, and ultrasound-based methods (19). NIBS is widely applied to modulate cortical excitability, producing facilitatory or inhibitory effects on various behaviors and functions (20). Studies indicate that combining NIBS with other interventions enhances cognitive and physical performance in older peoples with MCI (21). In particular, significant improvements have been reported when NIBS is integrated with physical activity programs (22). Additional research also supports the effectiveness of NIBS alone in improving cognitive function (23). Thus, the combined application of NIBS and cognitive stimulation represents a clinically replicable and promising strategy for managing mild cognitive impairment, as it integrates direct modulation of cortical excitability with functional activation of specific neural networks, enhancing memory, attention, and executive functions, while providing a safe and well-tolerated intervention in older peoples (24-26). Therefore, this systematic review with meta-analysis aimed to evaluate and synthesize the scientific evidence on interventions using NIBS combined with cognitive training on cognitive function in older peoples with MCI.

## 2 Methods

### 2.1 Protocol and registration

This systematic review and meta-analysis followed the methodologies outlined by the Cochrane Collaboration (27) and adhered to the PRISMA checklist and flowchart guidelines for reporting (28). The review protocol was registered in the PROSPERO database, CRD42024563219.

### 2.2 Eligibility criteria

This systematic review and meta-analysis included peer-reviewed original studies, specifically randomized controlled trials (RCTs), with no restrictions on language or publication date, up to May 2025. Studies were excluded if they were conference abstracts, books, book chapters, editorials, letters to the editor, protocol records, reviews, case studies, or non-randomized trials. The inclusion criteria were guided by the PICOS framework (Population, Intervention, Comparator, Outcome, Study design), as summarized in Table 1.

**Table 1:** Selection criteria used in the systematic review with meta-analysis.

Criteria	Inclusion criteria	Exclusion criteria
Population	Studies were included if they involved populations mean aged 60 years or older, with a diagnosis of MCI.	Studies with populations whose main pathology is other than MCI (i.e., chronic diseases, physical deterioration or social problems) and/or mean aged under 60 years.
Intervention	Studies involving NIBS combined with cognitive training in older people with MCI for 4 weeks or more.	Studies that include other types of complementary interventions, not related to NIBS.
Comparison	Interventions with active or inactive control groups.	Lack of baseline and/or follow-up data. Absence of control group.
Outcomes	At least one assessment of cognitive function.	Lack of baseline data and/or follow-ups.
Study design	Randomized controlled trials, with pre- and post-assessment.	Controlled, retrospective, prospective and cross-sectional, non-randomized studies.
Level of evidence	1a.	1b, 2a, 2b, 3a, 3b, 4 and 5.

MCI: mild cognitive impairment; NIBS: noninvasive brain stimulation.

### 2.3 Information and database search process

Seven databases were used: Medline/PubMed, Scopus, Cochrane, Web of Science (core collection), EBSCOhost, CINAHL, and ProQuest. Medical Subject Headings (MeSH) from the US National Library of Medicine and free-text phrases related to NIBS, cognitive function, older people, and MCI were used. The following search string was applied: (“Transcranial Magnetic Stimulation” OR “Magnetoencephalography” OR “Transcranial Direct Current Stimulation” OR “Electric Stimulation Therapy” OR “tDCS” OR “rTMS”) AND (“Executive Function” OR “Metacognition” OR “Attention” OR “Cognition” OR “Memory” OR “Problem Solving” OR “Decision Making” OR “Planning Techniques”) AND (“Aged” OR “older adults” OR “older people” OR “older subject” OR “aging” OR “ageing” OR “aged”) AND (“Cognitive Dysfunction” OR “Neurocognitive Disorders” OR “Cognitive Impairment Syndrome” OR “Early Cognitive Decline” OR “Mild Cognitive Changes” OR “Minor Cognitive Impairment”).

The included articles and the inclusion/exclusion criteria were reviewed by two independent experts with the following qualifications: (i) a Ph.D. in health-related sciences and (ii) peer-reviewed publications in journals with an impact factor (Journal Citation Reports®). The experts were not provided with the search strategy to minimize bias. A final database search on May 30, 2025, aimed to identify relevant errata or retractions related to the included studies.

An independent expert was consulted regarding the included articles and the application of the inclusion and exclusion criteria to ensure the identification of relevant studies. Two eligibility requirements were established for the expert: (i) holding a PhD in health sciences and (ii) having peer-reviewed publications in journals with an impact factor, according to Journal Citation Reports®, on topics such as virtual reality, quality of life, and cognitive function in older peoples. The search strategy was not disclosed to the expert in order to minimize bias. After these procedures were completed, the databases were searched again on August 30, 2025, to identify any relevant retractions or errata related to the included studies.

### 2.4 Study selection and data collection process

The studies were exported to Mendeley Reference Manager (version 2.116.1), and the selection process was documented in the PRISMA flowchart. Two authors (P. J.-O. and A. Q.) independently conducted the searches and systematically reviewed titles, abstracts, and full texts, while duplicates were removed. No discrepancies were identified at this stage. Potentially eligible articles were then re-examined, and exclusions were justified for studies that did not meet the predefined criteria. Finally, two additional reviewers (E. V.-C. and J. H.-M.) independently audited the entire selection and data extraction process.

### 2.5 Methodological quality assessing

The methodological quality and level of evidence were assessed using the Oxford Centre for Evidence-Based Medicine scale (29). Only level 1a studies, defined as RCTs, were included, while studies classified as levels 1b through 5 were excluded. RCTs were downgraded if concerns were identified regarding bias, consistency, accuracy, precision, or transparency of results (29).

### 2.6 Data collection process

Data from the included studies were extracted into a standardized form using Microsoft Excel® (version 16.81), in accordance with Cochrane guidelines (30). Two researchers (P. J.-O. and A. Q.) independently performed the extractions and compared their results to ensure accuracy, with oversight provided by a third reviewer (E. V.-C.). Extracted variables included authors, country, study design, sample size, group allocation (n), mean age, type of intervention and control condition, training volume (frequency, duration, intensity), type and intensity of NIBS, cognitive assessments, and main outcomes.

### 2.7 Risk of bias

The risk of bias in the included RCTs was assessed using the Risk of Bias 2 (RoB 2) tool (30). Two reviewers (P. J.-O. and A. Q.) conducted the initial assessment, which was subsequently reviewed by two additional authors (E. V.-C. and J. H.-M.). Discrepancies were resolved through discussion and consensus.

### 2.8 Meta-analysis measures

A meta-analysis approach was applied, with the detailed methodology registered in PROSPERO (CRD42024563219). For each comparison, the standardized mean difference (SMD) was calculated using Comprehensive Meta-Analysis software (RevMan 5.4). A *p*-value of <0.05 was considered statistically significant (31). A random-effects model, based on the DerSimonian–Laird method, was used to estimate and combine SMDs and mean differences across outcomes such as cognitive function, comparing experimental and control groups before and after the intervention (32). This model assumed that true intervention effects varied among studies due to factors such as intervention type or duration, thereby accounting for heterogeneity in effect sizes across populations.

Results were pooled when at least three studies reported consistent findings (33). Heterogeneity was assessed using the Cochrane Q test (34) and the I^2^ statistic, with thresholds of <25%, 25–50, and >50% representing low, moderate, and high inconsistency, respectively (32). Egger’s regression analysis was conducted to detect small-study effects and potential publication bias (35).

### 2.9 Certainty of evidence

The certainty of evidence from the included studies was evaluated using the Grading of Recommendations, Assessment, Development, and Evaluation (GRADE) framework (36). Evidence was categorized as high, moderate, low, or very low. All analyses initially started with a high certainty rating, given the inclusion of RCTs, but were downgraded if concerns arose regarding risk of bias, consistency, accuracy, precision, transparency of results, or publication bias. Two reviewers (P. J.-O. and A. Q.) conducted independent assessments, and disagreements were resolved through consensus with a third reviewer (E. V.-C.).

## 3 Results

### 3.1 Study selection

**Figure 1:**
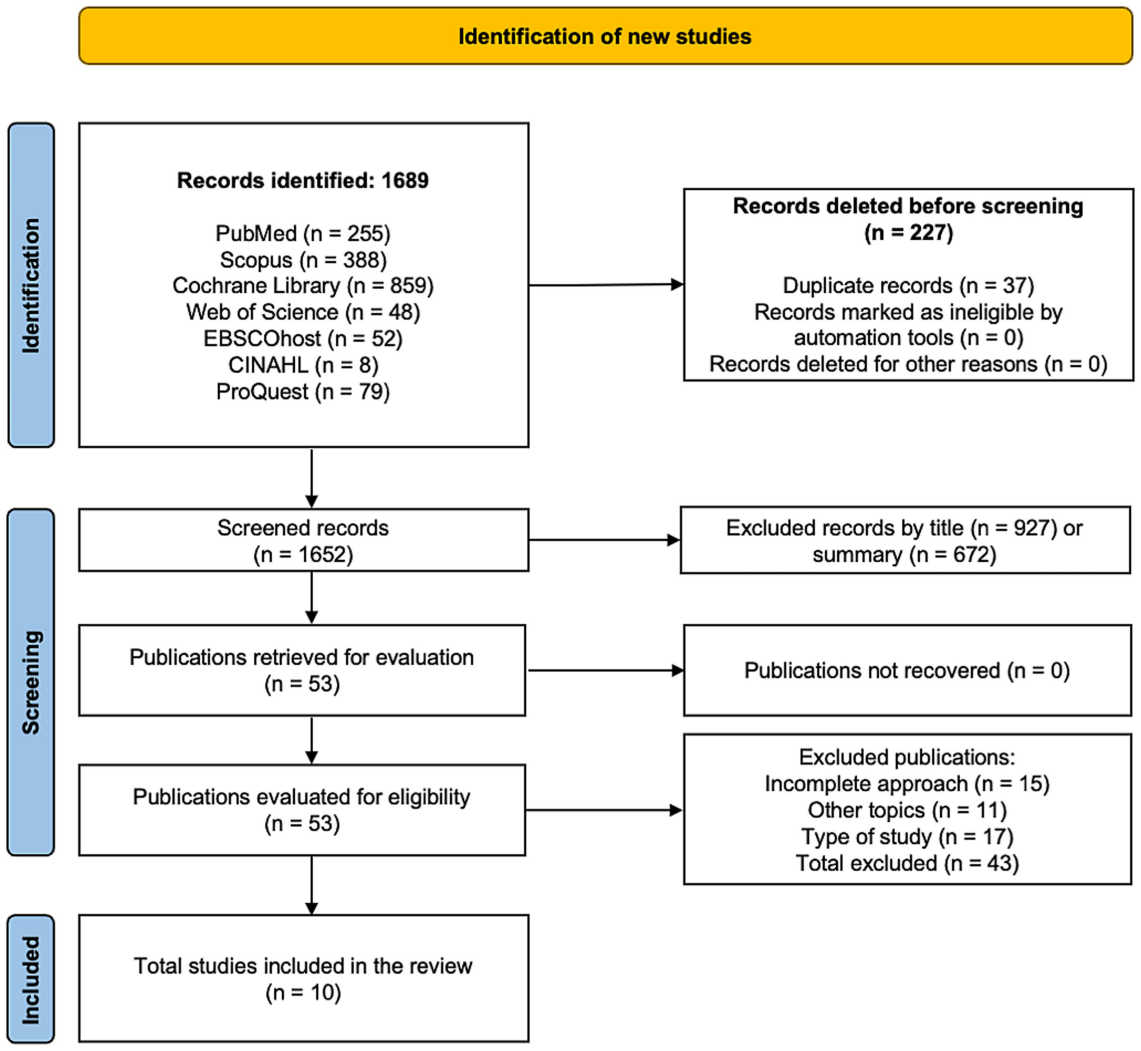
Flowchart of the systematic review.

A total of 1,689 studies were identified through the database search, with 37 excluded as duplicates. Of the remaining 1,652 records, 1,599 were excluded after screening titles and abstracts for relevance (927 based on titles and 672 based on abstracts). A full-text review was conducted for 53 references, of which 43 were excluded for not meeting the inclusion criteria: 15 due to incomplete approaches, 11 for unrelated topics, and 17 for not matching the required study design. Ultimately, 10 studies were included in the systematic review and meta-analysis (21, 37–45). The search and selection process is illustrated in a PRISMA flowchart (Figure 1) (46).

### 3.2 Methodological quality

The methodological quality of the included studies was rated as high. All 10 studies were randomized controlled trials (RCTs) (21, 37–45), representing the highest level of evidence (level 1a) according to the Oxford Scale. This study design minimized the risk of bias and provided a robust basis for evaluating the effectiveness of interventions involving NIBS combined with cognitive training in older peoples with MCI.

### 3.3 Risk of bias

**Figure 2:**
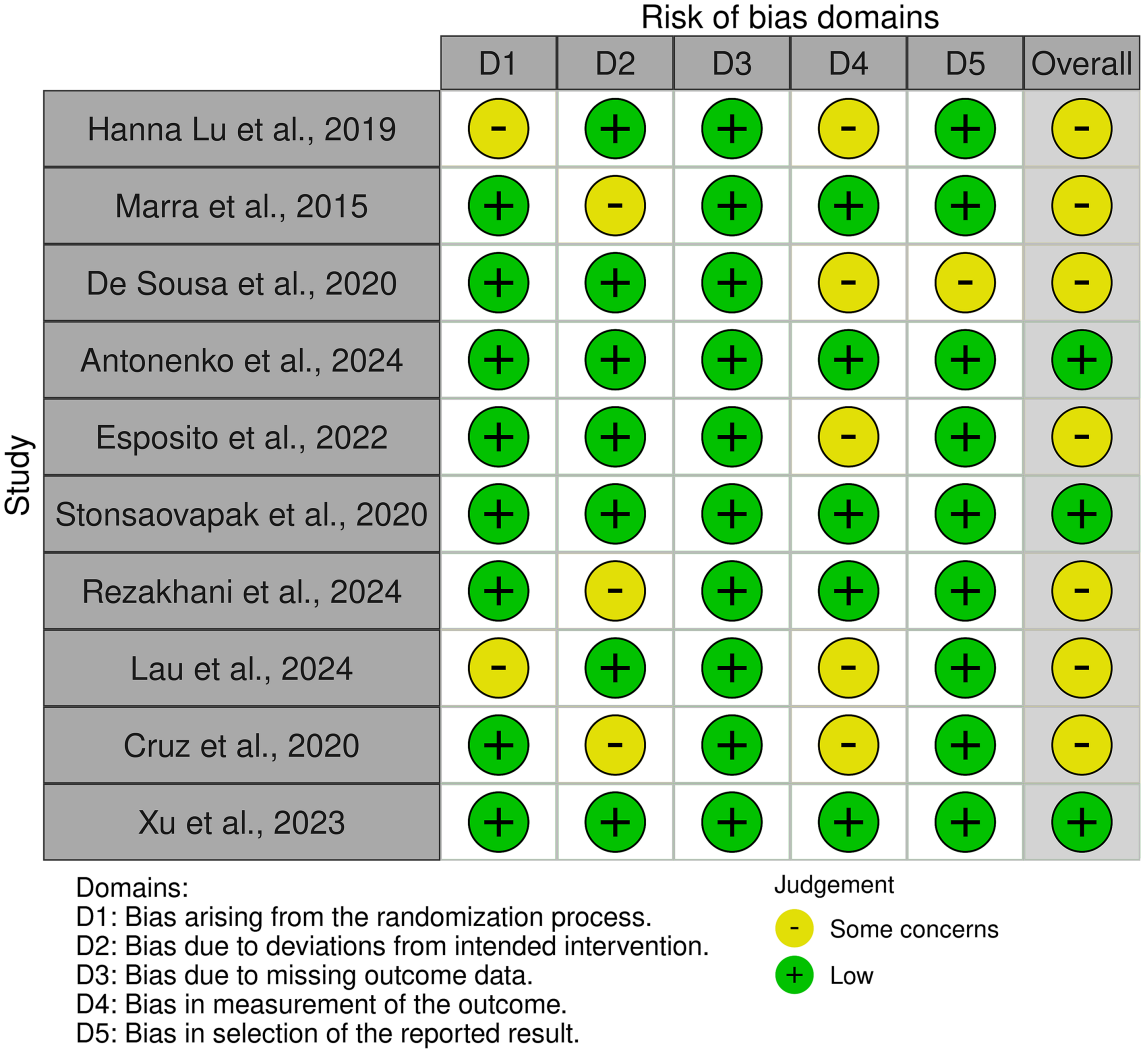
Risk of bias tool: traffic light chart.

**Figure 3:**
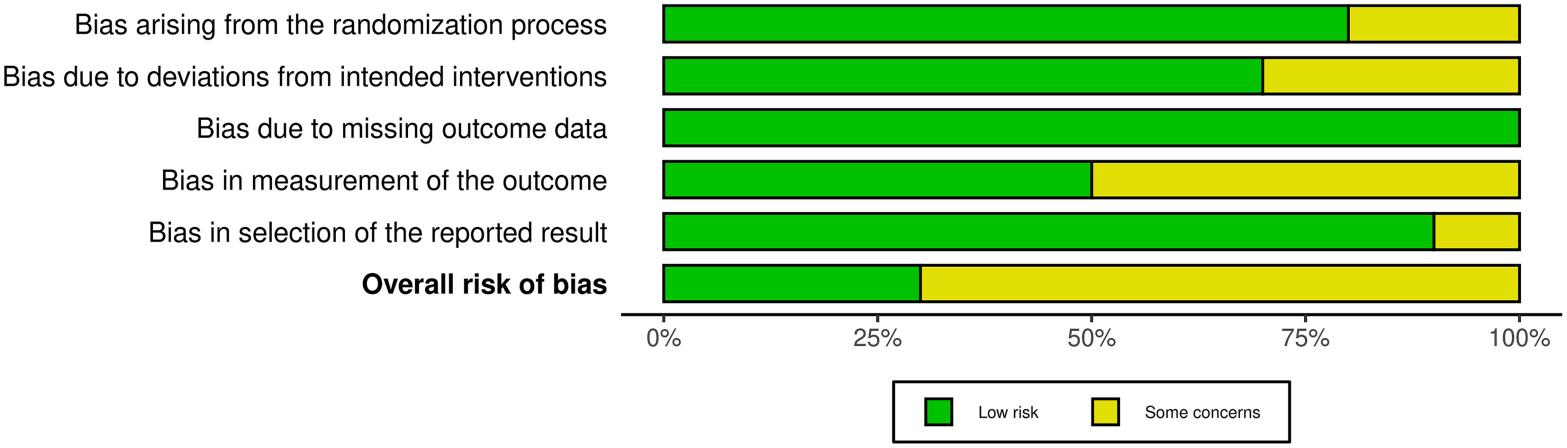
Risk of bias tool: summary table by domain.

Three studies were assessed as having a low risk of bias across all domains (39, 41, 45). Seven studies showed some concerns in one or more domains (21, 37, 38, 40, 42–44). None of the studies were classified as having a high risk of bias. Overall, the risk of bias was considered moderate, with most studies presenting some concerns and only a few demonstrating low risk across all domains. The risk of bias assessment is presented in Figures 2, 3.

### 3.4 Characteristics of the studies

The ten studies included in this review demonstrated considerable heterogeneity in interventions, participant characteristics, and cognitive outcomes. Interventions ranged from transcranial direct current stimulation (tDCS) combined with cognitive or physical training, to repetitive transcranial magnetic stimulation (rTMS) alone, and cognitive training without neurostimulation. Overall, tDCS particularly when paired with working memory, visuo-spatial tasks, or Tai Chi consistently enhanced memory, attention, and global cognitive scores, while rTMS improved verbal fluency and daily memory performance. Some studies reported task- or domain-specific effects, and minor improvements were occasionally observed in sham or control groups. Categorizing studies by intervention type and cognitive domain suggests that combining NIBS with cognitive training may provide clinically meaningful benefits for individuals with MCI, as summarized in Table 2.

**Table 2:** Selected studies on non-invasive brain stimulation combined with cognitive training in older people with mild cognitive impairment.

Author	Country	Sample (*n*)	Groups (*n*)	Mean age (y)	Training Volume			Type and Intensity of NIBS	Cognitive function	Main outcomes
					Week	Frequency (sessions/weeks)	Session duration (min)			
Langa and Levine (12)	CN	201	tDCS-WMT group (69); Sham tDCS-WMT group (64); tDCS-CCT Group (68)	73.4	4	5	45	tDCS 2 mA	MMSE, TMT-A, TMT-B, N back, Logical memory test	tDCS-WMT: ↑ Memory capacity (*p* < 0.001), fluency (*p* < 0.001), ↑ Delayed recall (*p* = 0.042) and N-back task performance (*p* = 0.04). Sham: Small ↑ memory capacity (*p* = 0.038) and N-back task performance (*p* = 0.024). tDCS-CCT: ↑ Cognitive function (*p* < 0.001), memory capacity (*p* < 0.001). tDCS-WMT > Sham (Memory); tDCS-WMT > tDCS-CCT (Cognition)
Senczyszyn et al. (25)	BR	34	rTMS (17); Sham (17)	65	2	5	20	rTMS, 10 Hz, 2000 pulses per day	RBMT, TMT-B	rTMS: ↑ Everyday memory (*p* = 0.029), sequencing (*p* = 0.029), verbal fluency (*p* = 0.036). Sham: No significant change (*p* > 0.05).
Pagali et al. (26)	CN	48	atDCS+Visuospatial (16); Sham (32)	70	4	3	30	tDCS 1 mA	MMSE, TMT-A, TMT-B, Boston Naming Test, Digit Span	No significant difference in training success (*p* = 0.74). atDCS: ↑ positive affect (day 3). No long-term memory change (details not specified)
Higgins et al. (27)	DE	39	atDCS (16); Sham (23)	70	4	3	45	tDCS 1 mA	N-back, AVLT, WMT2	No significant differences overall. atDCS: Small ↑ N-back (*p* = 0.06). Slightly higher performance at 7-month follow-up.
Page et al. (28)	IT	27	rTMS (11); Sham (16)	67.85	4	5	20	rTMS, 10 Hz, 2000 pulses	RBANS	rTMS: ↑ semantic fluency (*p* < 0.05), story memory (*p* < 0.05) & recall (*p* < 0.05). No baseline differences. ↑ MCI + TMS showed more pronounced apathetic symptoms compared to healthy controls
Manterola and Zavando (29)	TH	45	tDCS (23); Sham (22)	68.39	4	3	20	tDCS 2 mA	TMSE, MoCA, Singing task	No group differences in TMSE/MoCA. tDCS: ↑ RVP hits (p < 0.001), ↓ SWM errors, ↑ DMS hits (*p* < 0.001).
Higgins et al. (30)	IRN	60	tDCS-DLPFC (20); tDCS-DALT (20); Sham (20)	68.88	2	5	20	tDCS 2 mA	MoCA, QoLAD	tDCS-DLPFC & tDCS-DALT: ↑ MoCA at 2 weeks later (*p* ≤ 0.05), 1 month later (*p* ≤ 0.05), 3 months later (*p* ≤ 0.05). Sham: no change (*p* > 0.05). tDCS-DLPFC & tDCS-DALT: ↑ QoLAD at 3 months later (*p* = 0.001).
Verhagen et al. (31)	TW	21	tDCS+ICCT: (11); Sham+ICCT (10)	70	5	3	20	tDCS 2 mA	MoCA, TMT-A, TMT-B, N back, CVVLT	Both groups: ↑ Cognition (*p* < 0.001). tDCS+ICCT: Stronger effects on MoCA, TMT, CVVLT, N-back (*p* ≤ 0.05). Sham: Modest gains. No change in CDR.
Higgins et al. (32)	HK	67	tDCS+CT (22); Sham+CT (24); CT only (21)	69.8	3	3	30 min	tDCS 1.5 mA	MoCA, TMT-A, TMT-B, DST, RBMT-3	tDCS+CT: ↑ working memory (p ≤ 0.05), attention (time & reaction) (*p* ≤ 0.05). Sham: No decline reported.
Vásquez-Carrasco et al. (3)	CN	180	TCT (44); TCS (49); WAT (44); WAS (43)	60.25	12	3	60	tDCS 2 mA	MoCA, AVLT, Stroop Test	TCT, WAT or WAS: ↑ MoCA (*p* < 0.001), AVLT recall (*p* = 0.015), ↓ Stroop time after 12 weeks (*p* = 0.004).

atDCS: anodal transcranial direct current stimulation; AVLT: Auditory Verbal Learning Test; BR: Brazil; CCT: Cognitive Control Training; CDR: Clinical Dementia Rating; CN: China; CT: Cognitive training; CVVLT: Chinese Version of the Verbal Learning Test; DALT: Dorsolateral Anterior Left Temporal; DLPFC: Dorsolateral Prefrontal Cortex; DMS: Delayed Matching to Sample; DST: Digit Span Test; DE: Germany; HK: Hong Kong; ICCT: Integrated Cognitive and Cognitive Training; IRN: Iran; IT: Italy; MCI: Mild Cognitive Impairment; MMSE: Mini-Mental State Examination; MoCA: Montreal Cognitive Assessment; N back: Working Memory Test; NIBS: Non-Invasive Brain Stimulation; QoLAD: Quality of Life in Alzheimer’s Disease; RBANS: Repeatable Battery for the Assessment of Neuropsychological Status; RBMT: Rivermead Behavioral Memory Test; RBMT-3: Rivermead Behavioral Memory Test 3; rTMS: Repetitive Transcranial Magnetic Stimulation; RVP: Rapid Visual information Processing; SWM: Spatial Working Memory; TH: Thailand; TCT: Tai Chi Combined with tDCS; TCS: Tai Chi Combined with Sham tDCS; tDCS: transcranial direct current stimulation; TMT-A: Trail Making Test Part A; TMT-B: Trail Making Test Part B; TMSE: Thai Mental State Examination; tDCS: Transcranial Direct Current Stimulation; TW: Taiwan; WAS: Walking Combined with Sham tDCS; WAT: Walking Combined with tDCS; WMT: Working Memory Training.

### 3.5 Sample characteristics

The total population included in this systematic review and meta-analysis comprised 722 older peoples, of whom 71.4% were female, with a mean age of 69.5 years. Sample sizes ranged from 22 participants (43) to 201 participants (15), reflecting variability in study design and intervention scope. The interventions assessed included tDCS combined with cognitive training, repetitive rTMS, and cognitive training alone, each evaluated for their effects on cognitive function in older peoples with MCI.

### 3.6 Doses and interventions performed

The included studies implemented a range of NIBS and cognitive interventions aimed at improving cognitive function in individuals with MCI. Cognitive training programs, such as memory and attention tasks combined with neurostimulation techniques like tDCS and rTMS, primarily targeted cognitive performance (21, 37, 43). Individualized interventions combining physical activity and cognitive training, such as Tai Chi or walking paired with tDCS, also demonstrated significant improvements in cognitive outcomes (44, 45). Intervention protocols varied in duration and frequency, ranging from 4 weeks with three to five sessions of 20–45 min per week (21, 37, 43) to 12 weeks with multiple 30–60-min sessions (45). Most programs were conducted at moderate intensity, with tDCS doses between 1 and 2 mA (21, 43).

### 3.7 Cognitive function

The overall effects of NIBS on cognitive function variables are summarized in Table 3, with corresponding forest plots presented in Supplementary Figures 1–3. Significant moderate effects (*p* < 0.05) were observed for Trail-Making Test Part A (TMT-A; ES = 0.54) and MoCA (*p* > 0.05), indicating that NIBS preferentially enhances processing speed, attention, and global cognitive performance. In contrast, TMT-B showed no significant differences, with small to moderate effect sizes (ES = 0.05–0.52). These differential outcomes may reflect the varying sensitivity of cognitive measures to NIBS: TMT-A and MoCA primarily assess domains such as sustained attention, executive control, and working memory, which are more directly influenced by cortical excitability and network plasticity modulated by NIBS. Conversely, TMT-B, which imposes greater demands on cognitive flexibility and task-switching, may require more extensive or targeted interventions to elicit measurable improvements. Considering these distinctions enhances the interpretative rigor and underscores the importance of selecting appropriate outcome measures aligned with the neurophysiological mechanisms targeted by NIBS.

**Table 3:** Effects of NIBS combined with cognitive training on cognitive function in older people with mild cognitive impairment.

Tests	*n* of studies	*n* of experimental groups	*n* of control groups	Total participants	ES (95%CI)	*p*	*I*^2^ (%)	Egger’s test (*p*)	RW (%)
MoCA	5	5	5	277	0.52 (0.34 to 1.17)	0.05	76.6	0.00	7.21–8.53
TMT-A	4	4	4	294	0.54 (0.003 to 1.08)	0.04	76.6	0.00	21.8–25.2
TMT-B	5	5	5	328	0.05 (−0.35 to 0.45)	0.80	65.6	0.02	−0.93-11.6

CI: confidence interval; ES: effect size; MoCA: Montreal Cognitive Assessment; RW: relative weight; TMT-A: Trail Making Test Part A; TMT-B: Trail Making Test Part B.

### 3.8 Certainty of evidence

The available evidence is not robust enough to provide definitive recommendations for interventions targeting cognitive function in older people with MCI. While certain studies have shown promising results, the overall findings highlight the necessity for further research to draw clearer conclusions and develop evidence-based strategies for this population (Table 4).

**Table 4:** Assessment of methodological quality using the GRADEpro tool.

Certainty of evidence							*N* of patients		Effect		Certainty	Importance
*N* of studies	Study design	Risk assessment	Inconsistency	Indirect evidence	Vagueness	Other considerations	NIBS and cognitive training	Active control group	Relative (95% CI)	Absolute (95% CI)		
MoCA (Montreal Cognitive Assessment)												
5	RCT	Serious	It’s not serious	It’s not serious	It’s not serious	None	120/237 (50.6%)	117/237 (49.4%)	Not estimable		+++ Moderate	IMPORTANT
TMT (Trail Making Test) A												
3	RCT	Serious	It’s not serious	It’s not serious	It’s not serious	None	86/175 (49.1%)	89/175 (50.9%)	Not estimable		+++ Moderate	IMPORTANT
TMT (Trail Making Test) B												
4	RCT	Serious	It’s not serious	It’s not serious	It’s not serious	None	101/209 (48.3%)	108/209 (51.7%)	Not estimable		+++ Moderate	IMPORTANT

CI: confidence interval; NIBS: noninvasive brain stimulation; RCT: randomized clinical Trial.

### 3.9 Adverse effects and adherence

The studies analyzed in this systematic review with meta-analysis indicated good participant adherence (87.7%) and did not mention any adverse effects. This implies that the interventions were generally well tolerated and practical for older people with MCI, highlighting their potential for wider application in similar populations.

## 4 Discussion

### 4.1 Cognitive functions MoCA

The meta-analysis revealed significant moderate effects (*p* < 0.05) supporting the effectiveness of both NIBS and cognitive training, as evaluated by the MoCA. No significant differences were observed in baseline MoCA scores between active and sham groups in a randomized controlled trial investigating the effects of tDCS combined with working memory training in older peoples with MCI (*p* > 0.05) (47). Draaisma et al. (48) reported that in a cohort of 20 healthy older peoples, personalized theta transcranial alternating current stimulation (tACS) significantly improved performance in a motor sequence learning task with a high working memory load (*p* < 0.001), as well as task speed (p < 0.001) and accuracy (*p* = 0.03). Similarly, a study involving 229 healthy participants demonstrated substantial improvements in inhibition and cognitive flexibility after both tACS and tDCS, with no significant differences between the two methods. Functional near-infrared spectroscopy showed that tDCS reduced functional connectivity in relevant cortical regions, suggesting that transcranial stimulation enhances executive function, with tDCS conferring superior neural benefits compared to tACS (49). Additionally, another study reported cognitive improvements following 12 sessions of either active or sham tDCS in older peoples, with the tDCS plus sertraline group exhibiting a significant increase in MoCA scores (*p* = 0.01) (50). NIBS targets cortical excitability, modulating neuronal activity and promoting the formation of new synaptic connections, repeated and spaced-out application of stimuli has been shown to be more effective in inducing lasting changes in synaptic plasticity, a principle that aligns with the mechanisms of learning and memory in the human brain (51). Furthermore, NIBS has shown potential in the treatment of neurocognitive disorders by improving neuronal connectivity and restoring altered cognitive functions (52).

### 4.2 Cognitive functions TMT

The meta-analysis revealed significant moderate effects (*p* < 0.05) in favor of NIBS combined with cognitive training for TMT-A (*p* = 0.04). In contrast, no significant differences were found for TMT-B. One RCT reported that the combination of tDCS and Nintendo Switch significantly improved performance on both TMT-A (*p* = 0.03) and TMT-B (*p* = 0.04) in individuals with chronic stroke, suggesting that interactive training may enhance the effects of brain stimulation (53). Similar findings were reported in an RCT with 103 participants diagnosed with depression who received tDCS, where a significant improvement in TMT-A performance was observed (*p* ≤ 0.02), indicating enhanced processing speed (49). However, a placebo-controlled RCT involving 25 patients with Alzheimer’s disease, in which tDCS was applied to the left temporal cortex over six sessions, reported no significant effects on either TMT-A (*p* = 0.378) or TMT-B (*p* = 0.093) (54). Overall, the findings suggested that NIBS combined with cognitive training improved certain cognitive abilities, particularly processing speed and general cognitive performance, as reflected in TMT-A and MoCA scores. However, its effects on more complex executive tasks, such as those measured by TMT-B, remained less clear. Research using tDCS and tACS has shown promising benefits for executive function and working memory, and plasticity though results vary depending on the specific task and the population studied (55).

### 4.3 Limitations and strengths

Limitations include: (i) The relatively small number of included studies reduces statistical power and robustness of conclusions; (ii) Substantial heterogeneity in intervention protocols complicates direct comparisons; (iii) Evidence certainty was rated as low to moderate, with methodological concerns in most trials and short follow-up periods (≤3 months); (iv) Generalizability is limited by geographical concentration and sample imbalances; (v) High equipment costs may hinder accessibility in community settings; (vi) specialized training requirements could restrict implementation, and (vii) biological variability (e.g., cranial anatomy) may influence stimulation efficacy.

Strengths include: (i) Exclusive inclusion of RCTs enhanced methodological rigor and reliability of findings; (ii) Multiple neurostimulation modalities, including tDCS and rTMS, were evaluated in combination with cognitive training, providing a comprehensive assessment; (iii) Significant cognitive benefits were identified in domains such as working memory, processing speed, and verbal fluency, suggesting promise for improving cognitive function in older peoples with MCI; and (iv) The review offers practical insights for future research and potential population-level implementation strategies.

### 4.4 Practical applications

The moderate effect sizes observed for cognitive outcomes such as TMT-A and MoCA highlight the innovative potential of combining NIBS with targeted cognitive exercises for older peoples with MCI. This approach represents a scalable and practical strategy for enhancing cognitive function within community or outpatient settings. Specifically, pairing low intensity tDCS (1–2 mA) with working memory or verbal fluency training can be delivered safely in small groups, with high adherence (87.7%) and no reported adverse events. The clinical significance lies in its feasibility: interventions can be administered by allied health professionals with minimal equipment, guided by standardized manuals detailing electrode placement, session timing (20–45 min, 3–5 sessions/week), and progression criteria. Furthermore, digital platforms offer opportunities for remote supervision, real-time data collection, and participant engagement, supporting broader implementation and integration into routine care. Overall, this combined approach provides a replicable, low-risk, and evidence-based pathway for translating cognitive enhancement research into practical clinical applications.

### 4.5 Clinical applications

Clinicians in geriatric services, or neurorehabilitation settings may consider incorporating NIBS combined with cognitive training as an adjunct to conventional pharmacological and behavioral therapies. Evidence from RCTs demonstrating improvements in processing speed and memory capacity (31, 33) highlights the potential of these interventions for individuals with early MCI. Clear patient selection criteria including baseline MoCA scores, comorbidities, and cranial anatomy are critical to optimize efficacy and reduce variability. Standardized assessments at baseline, mid-intervention, and follow-up can guide personalized adjustments. Successful integration of NIBS protocols will require interdisciplinary collaboration among neurologists, psychiatrists, neuropsychologists, and rehabilitation specialists to monitor safety, ensure adherence, and evaluate long-term functional outcomes such as daily living activities and quality of life.

### 4.6 Epidemiological applications

At a population level, NIBS-based cognitive training has the potential to slow MCI progression and reduce dementia incidence among older peoples. Epidemiological modeling incorporating effect size estimates can quantify potential reductions in disability-adjusted life years (DALYs) and healthcare costs. However, the high cost of equipment may pose barriers in community-based settings, requiring reimbursement strategies and scalable implementation frameworks to ensure accessibility across socioeconomic groups. In addition, specialized training and technical expertise are essential for safe and effective administration, which may limit availability in under-resourced health systems. Biological variability, particularly differences in cranial anatomy, can further influence stimulation efficacy, highlighting the need for individualized approaches and adaptive protocols. Large-scale, population-level studies are warranted to assess the durability of cognitive benefits and safety in real-world settings. Integration into national aging policies through community health programs, clinician training, and equitable funding models could strengthen preventive efforts, inform evidence-based guidelines for MCI management, and support sustainable cognitive health interventions at the societal level.

## 5 Conclusion

The combination of NIBS and cognitive training interventions demonstrated significant improvements in cognitive functions in older people with MCI, particularly in tasks like TMT-A and MoCA where moderate effects were observed. However, no significant differences were found in TMT-B scores, indicating that while the interventions may benefit some cognitive processes, their impact on others remains uncertain.

## Data Availability

The original contributions presented in the study are included in the article/Supplementary material, further inquiries can be directed to the corresponding authors.
