# Supplementary material for: Effects of non-invasive brain stimulation combined with cognitive training on cognitive functions in older people with mild cognitive impairment: a systematic review with meta-analysis
Source: Front Med (Lausanne). 2025 Oct 9;12:1659208. doi: 10.3389/fmed.2025.1659208 (PMC12546134; doi:10.3389/fmed.2025.1659208)
Supplement: Supplementary file 1 [file Data_Sheet_1.docx]

Supplementary Material

## Supplementary Figures


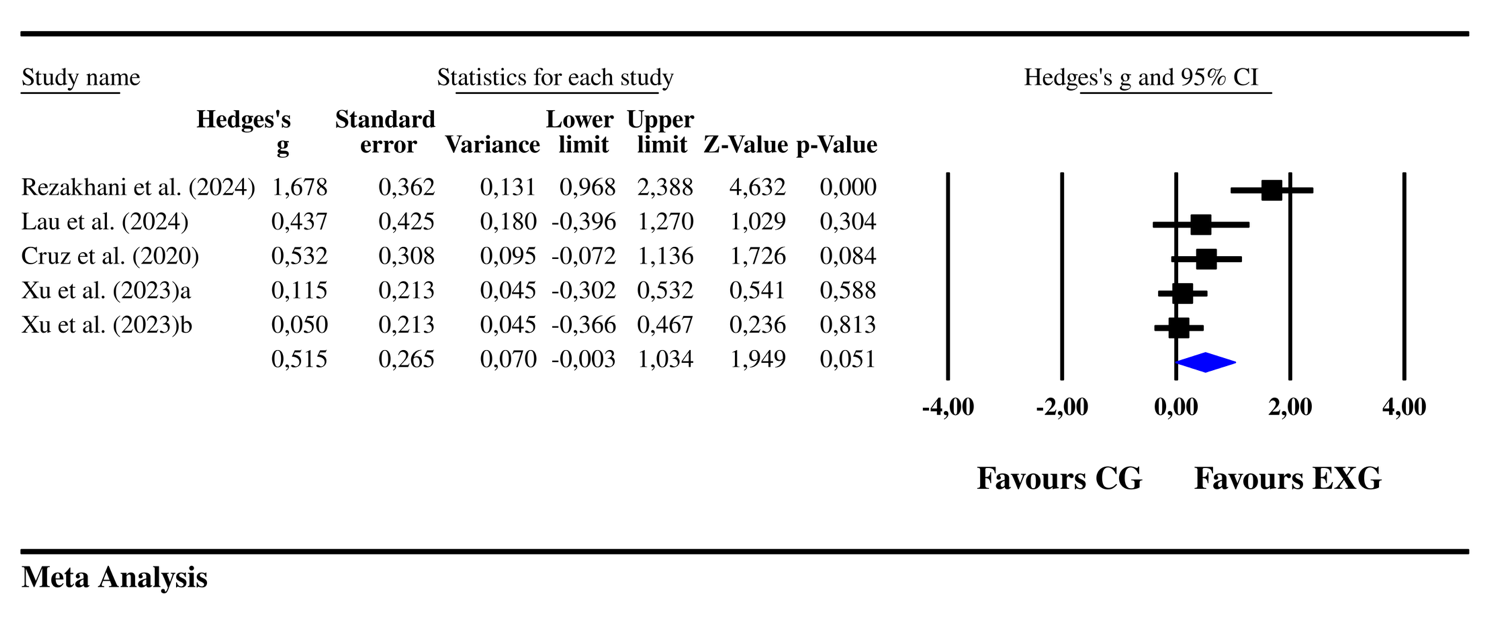


**Supplementary Figure 1.** Forest plot of changes in MoCA in favor of NIBS and cognitive training participating in 120 compared with 117 assigned as controls. Values shown are effect sizes (Hedges' g) with 95% confidence intervals (CI). The size of the squares plotted reflects the statistical weight of each study.

**
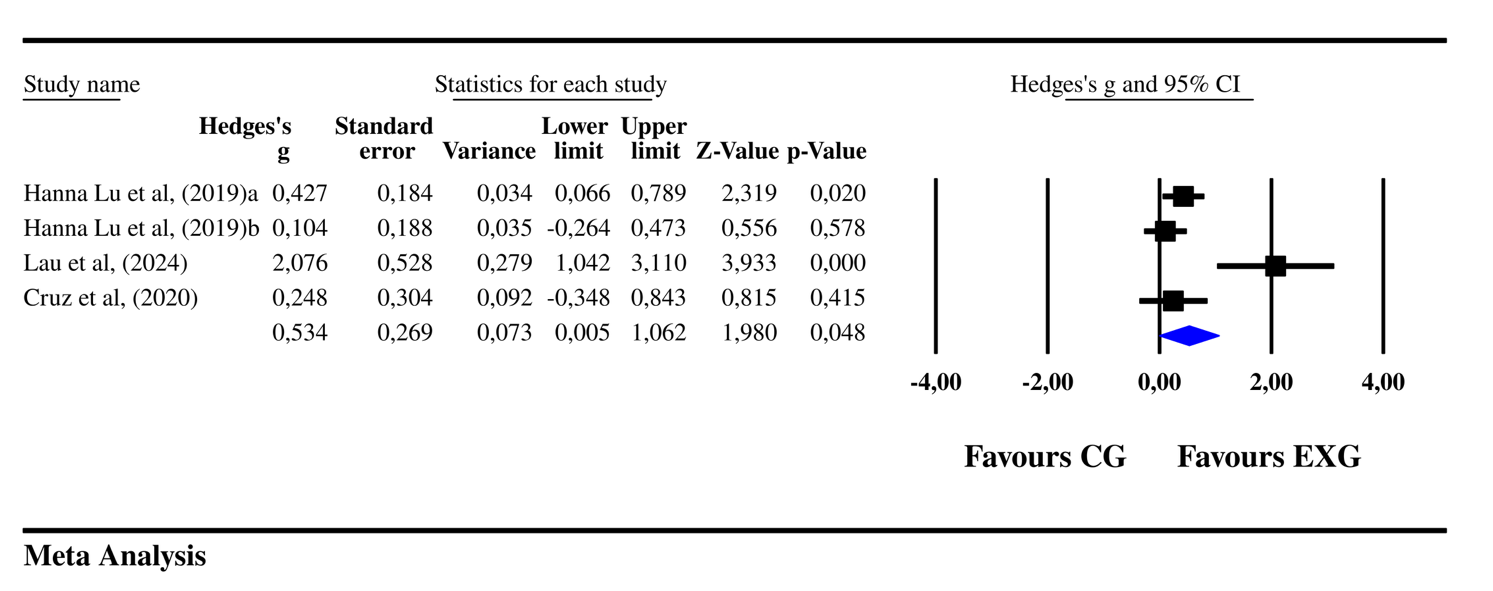
**

**Supplementary Figure 2.** Forest plot of changes in TMT-A in favor of NIBS and cognitive training participating in 86 compared with 89 assigned as controls. Values shown are effect sizes (Hedges' g) with 95% confidence intervals (CI). The size of the squares plotted reflects the statistical weight of each study.

**
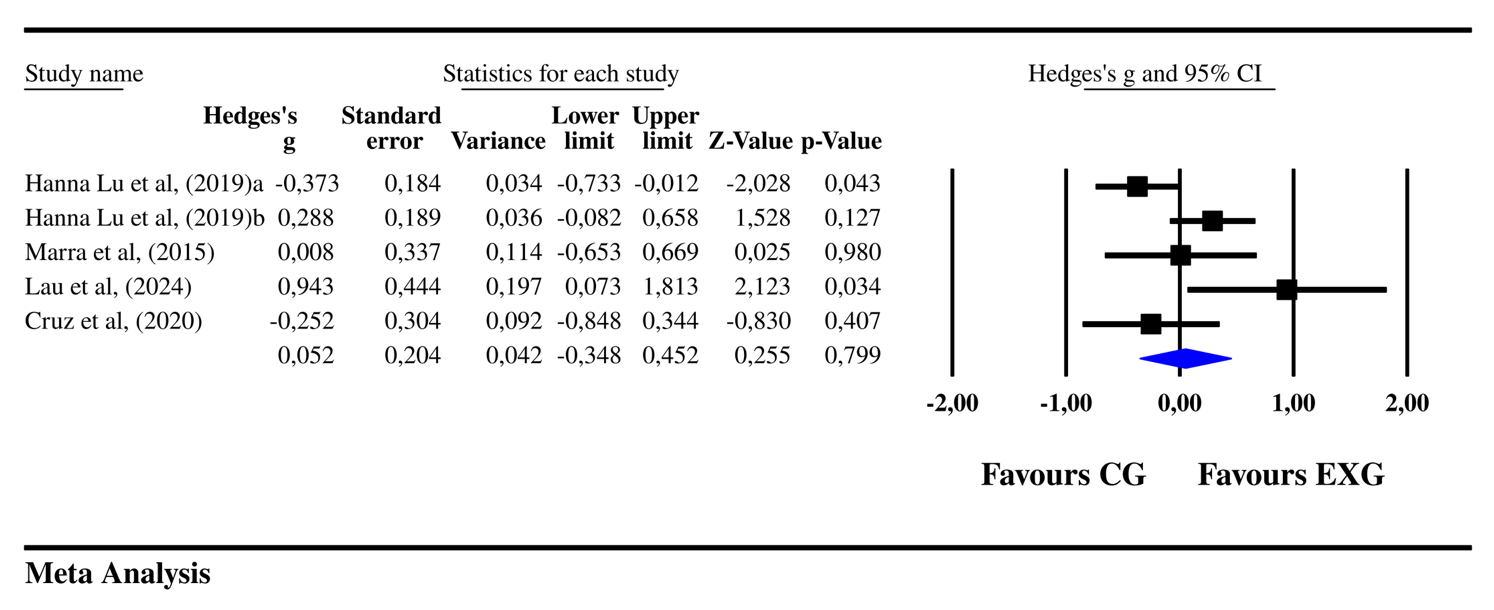
**

**Supplementary Figure 3.** Forest plot of changes in TMT-B in favor of NIBS and cognitive training participating in 101 compared with 108 assigned as controls. Values shown are effect sizes (Hedges' g) with 95% confidence intervals (CI). The size of the squares plotted reflects the statistical weight of each study.
